# Deep Learning for Predicting Spheroid Viability: Novel Convolutional Neural Network Model for Automating Quality Control for Three-Dimensional Bioprinting

**DOI:** 10.3390/bioengineering12010028

**Published:** 2025-01-01

**Authors:** Zyva A. Sheikh, Oliver Clarke, Amatullah Mir, Narutoshi Hibino

**Affiliations:** 1 Section of Cardiac Surgery, Department of Surgery, University of Chicago, 5841 S. Maryland Ave., Chicago, IL 60637, USA; zyvasheikh@uchicago.edu (Z.A.S.); oliverclarke@uchicago.edu (O.C.); amir1@uchicago.edu (A.M.); 2 Pediatric Cardiac Surgery, Advocate Children’s Hospital, 4440 W 95th St., Chicago, IL 60453, USA

**Keywords:** spheroid, deep learning, 3D-bioprinting, viability, prediction, convolutional neural networks, tissue biofabrication

## Abstract

Spheroids serve as the building blocks for three-dimensional (3D) bioprinted tissue patches. When larger than 500 μm, the desired size for 3D bioprinting, they tend to have a hypoxic core with necrotic cells. Therefore, it is critical to assess the viability of spheroids in order to ensure the successful fabrication of high-viability patches. However, current viability assays are time-consuming, labor-intensive, require specialized training, or are subject to human bias. In this study, we build a convolutional neural network (CNN) model to efficiently and accurately predict spheroid viability, using a phase-contrast image of a spheroid as its input. A comprehensive dataset of mouse mesenchymal stem cell (mMSC) spheroids of varying sizes with corresponding viability percentages, which was obtained through CCK-8 assays, was established and used to train and validate the model. The model was trained to automatically classify spheroids into one of four distinct categories based on their predicted viability: 0–20%, 20–40%, 40–70%, and 70–100%. The model achieved an average accuracy of 92%, with a consistent loss below 0.2. This deep-learning model offers a non-invasive, efficient, and accurate method to streamline the assessment of spheroid quality, thereby accelerating the development of bioengineered cardiac tissue patches for cardiovascular disease therapies.

## 1. Introduction

Tissue engineering integrates the materials science and quantitative methods of biomedical engineering with the stem cell biology and physiological principles of regenerative medicine [1]. Originally, the concept of cellular therapy dominated, in which cells are injected into patients for medical treatments [1]. However, tissue engineering arose for cases in which the diseased site was a three-dimensional (3D) bulky and complex tissue structure for which cell injections were ineffective in treating [1]. The extracellular matrix (ECM), which influences several aspects of cell behavior, is damaged or lost in most diseases and injuries, so the injected cells still receive abnormal ECM cues [2]. Therefore, biomaterial scaffolds have been deemed necessary to facilitate a new microenvironment that mimics the original healthy ECM and provides cues that promote tissue repair or regeneration from the infiltrating cells [2].

Furthermore, the rising demand for organ transplants points toward tissue engineering as the solution to the organ shortage crisis and the immune rejection that follows transplantations. Tissue engineering aims to “create cell-scaffold constructs to direct tissue regeneration and to restore function through the delivery of living elements, which become integrated into the patient” [1]. For example, our research in cardiac tissue engineering strives to develop cardiac tissue patches that mimic the structure and function of native heart tissue to enhance in situ myocardial regeneration following myocardial infarction, thereby alleviating the need for a heart transplant [3]. Other researchers are developing retinal patches to treat age-related macular degeneration and decellularized 3D liver scaffolds to engineer a whole liver [4,5].

Porous 3D biomaterial scaffolds serve as an ECM-like guiding framework for cells to adhere to, expand, differentiate, and produce matrices for neotissue formation [1]. The cells in these scaffolds are often differentiated cells reprogrammed into undifferentiated stem cells, known as induced pluripotent stem cells (iPSCs). The iPSCs can then be differentiated into a new desired cell type, such as cardiomyocytes or pancreatic beta cells, to develop into a bioengineered tissue patch. Patient-derived iPSCs facilitate a decreased risk of immune rejection since the cells used to create the tissue patch are native and genetically similar to the patient. The scaffolds are seeded with these cells and are either cultured in vitro to form tissues that can be implanted into an injured site or are implanted directly into the injured site to leverage the body’s systems to induce regeneration of tissues or organs in vivo [6]. To further replicate ECM properties in biomaterial scaffolds, growth factors can be added to trigger differentiation into distinct cell lineages, and peptide sequences with bioactive roles can be used to functionalize synthetic polymers [7,8]. Additionally, the stiffness of the ECM-like scaffold plays a role in cell migration behavior and in inducing cell differentiation [7].

Three-dimensional cell cultures have played a pivotal role in the field of tissue engineering. Two-dimensional (2D) cultures fail to adequately recapitulate the 3D in vivo environment and lack cell-to-cell and cell-to-ECM interactions, inhibiting cells from maintaining their normal features and behaviors [9]. Conversely, 3D cultures are more physiologically relevant to in vivo conditions and allow for the development of 3D tissue models for the preclinical testing of new therapeutics or engineered tissue patches [10]. Muguruma et al. found that, in triple-negative breast cancer cell lines, 3D culture was more resistant to anticancer drugs than 2D; this resistance is also observed in vivo, demonstrating the need for 3D cultures [11].

Spheroids are three-dimensional (3D) multicellular aggregations of one or more cell types, ranging in diameter from 50 to 1000 μm. They tend to have increased cell survival rates, higher levels of ECM protein secretion, and a more stable morphology, compared to 2D culture [12,13]. Spheroids have been traditionally used in cancer research for understanding the pathophysiology of cancer progression and resistance, conducting in vitro screening of anticancer treatments, and reproducing in vitro the specificity of a patient’s tumor to personalize screening of the most effective treatments [14]. The optimum size for these spheroids is ~100,350 μm as larger spheroids may not be suitable for studying drug toxicity and other biological parameters [15]. They enhance the predictive power and reduce both time and financial costs during the later stages of the drug development timeline, allowing for the early detection of ineffective agents and reducing the risk of drug withdrawal from the market [14].

Spheroids have more recently been combined with 3D bioprinting, allowing for the production of tissue patches without scaffolding. Three-dimensional bioprinting has made depositing cells or spheroids easier, faster, more accurate, and more reproducible than by hand [16]. For example, the S-PIKE bioprinter leverages the Kenzan method, or microneedle-based method, by stacking the spheroids on stainless-steel needle arrays that temporarily support the spheroids and allow in situ fusion of the spheroids to form a cardiac macro-tissue [12]. Once the cardiac tissue is formed, the needle arrays are retracted, and the tissue can be perfused and cultured for further maturation [12]. Scaffold-free biofabrication is an appealing approach as the lack of non-native biomaterials better resembles native tissue, and the self-assembly is similar to physiological conditions. It has also been suggested that cell differentiation, cell-to-cell interactions, remodeling, and assembly occur faster as there is no physical barrier for cells to overcome in a scaffold-free structure [16]. However, achieving complex and large architectures may be more technically challenging due to the lack of support and shaping from scaffolding [16]. Scaffold-free biofabrication can also be relatively expensive and time-consuming when spheroids are manually made.

**Figure 1:**
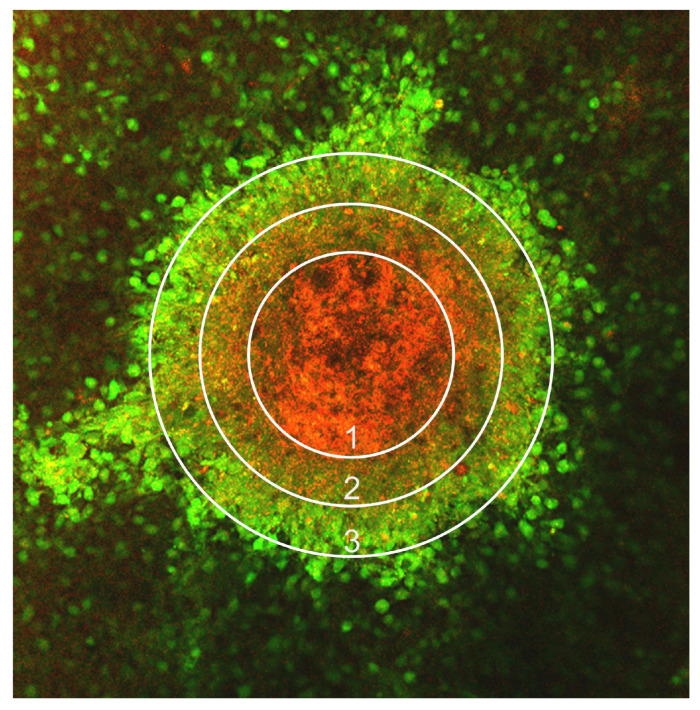
Image of a spheroid made with 33 k cells (20% mMSCs and 80% mouse endothelial cells) stained with calcein-AM (green) and ethidium homodimer-1 (red), which was captured on the STELLARIS confocal microscope: (1) Indicates the hypoxic core; (2) Indicates the inactive layer; (3) Indicates the proliferating layer.

Due to the fixed distance of ~400 μm between Kenzan needles, the size-consistent spheroids must have a diameter of ~600 μm to ensure direct contact [12]. However, transport limitation phenomena in spheroids larger than ~250 μm in diameter diminish the diffusion of oxygen and nutrients into their cores, resulting in severe hypoxia and tissue necrosis [10,12]. Therefore, an increase in spheroid size negatively correlates to overall viability. An ordered gradient of proliferation rates is observed in spheroids larger than 500 μm, with a proliferative zone at the surface due to abundant oxygen and culture medium, a quiescent and normoxic zone in the middle, and a necrotic and hypoxic zone at the center core Figure 1 [12]. The hypoxic cells anaerobically convert pyruvate to lactic acid and produce an acidic core within spheroids Figure 1 [12]. To develop tissue patches that will ultimately be implanted into patients, the patch’s spheroids must maintain high cell viability. Therefore, mitigating this hypoxic core with necrotic cells is unique to our applications as we scale up in spheroid size, and it requires innovative solutions.

It is critical to assess spheroid viability to ensure that the spheroids are suitable for 3D bioprinting to create tissue patches. In this paper, viability refers to the ratio of healthy living cells to the total number of cells, which can be determined using several assays. Live/Dead viability assays use fluorescent dyes to distinguish between live and dead cells. To do so, the spheroids must be dissociated into a single-cell solution. Live cells are typically stained with a green calcein-AM fluorescent dye, and dead cells are stained with a red ethidium homodimer-1 fluorescent dye. Fluorescence microscopy can be used for a qualitative assessment, and fluorescence-activated cell sorting (FACS) on a flow cytometer can quantify the numbers of living and dead cells. However, using the flow cytometer and FlowJo 10.10, which analyzes and interprets FACS data, often requires specialized training. Using a spectrometer, colorimetric assays such as MTT and Cell Counting Kit-8 (CCK-8) measure the metabolic activity of cells by quantifying the color change from the conversion of tetrazolium salts into formazan products by metabolically active cells. These assays are faster and less complex than FACS, but they still require several hours to complete. The TUNEL assay detects apoptotic cells by labeling DNA strand breaks with fluorescently labeled nucleotides, which are incorporated into the free 3′-OH ends of fragmented DNA by terminal deoxynucleotidyl transferase (TdT). Typically, it requires three or more hours to complete TUNEL staining. Overall, these viability assays are intensive and require significant manual labor and time. Furthermore, they require the addition of reagents, which have the potential to damage cells and can leave the spheroids unsuitable for further applications. As a result, there is a vital need to establish an accelerated and non-invasive method of assessing the viability of spheroids to streamline the production of high-viability tissue patches.

The predictive capacity of machine learning has been powerful in automating otherwise time-consuming and labor-intensive tasks, especially in biomedical research [17,18]. Deep learning (DL), a subset of machine learning inspired by the human brain, is an attractive tool for generating our desired method. DL uses multi-layered neural networks to automatically extract features from data to learn intricate patterns and representations. Convolutional neural networks (CNNs) are a key architecture in DL for image recognition and classification. It involves supervised learning, which uses labeled training datasets to train algorithms to recognize patterns and predict outcomes [19]. Kim et al. developed a CNN model that could distinguish differences in cell morphologies for predicting stem cell state [20]. Similarly, Benning et al. leveraged CNNs to predict the effect of drug exposure on spheroids [21]. There has also recently been a significant push toward implementing DL specifically for 3D bioprinting applications [22,23,24,25,26]. We hypothesize that if there is a CNN model that can accurately predict the percent viability of spheroids, then the production of viable cardiac tissue patches can be expedited. We aim to harness the CNN model’s capacity to discern intricate patterns in spheroid diameter, cell density, and circularity, ultimately exploiting their correlations with spheroid viability to yield accurate predictions.

CNN models are made of four main layers: a convolutional layer, a rectified linear unit layer (ReLU), a pooling layer, and a fully connected (FC) layer. These layers can be implemented multiple times, resulting in tens or hundreds of layers that learn to detect image features. Convolutional layers apply a filter known as a kernel to read a part of an image. Then, it forms a conclusion as an array of numbers, multiplies the array, and deduces a single number [27]. The kernel moves right by one unit and repeats this process until it has read the entire image, assigning a single number to each unit stored in a 3D array [27]. The convolutional layer essentially processes the input image and generates an activation map, which is a visual representation of the image that shows the kernel’s response to each spatial position of the image [28]. The output is then processed by the ReLU, which is an activation function that concludes the output of the neural network [28]. The activation function maps the output values between zero and one; it outputs zero if it receives a negative input but returns that value if it receives a positive input [28]. The pooling layer is responsible for reducing the size of activation maps and ignores less significant data while still preserving the most dominant features in each pool step [27]. The advantages of this dimensionality reduction are threefold: less computational power is required to process the data, time can be saved on training, and overfitting—whereby the model memorizes the training data and fails to generalize to new data—can be prevented [28]. The CNN models in this paper utilize maximum pooling, which selects the maximum value for each patch within the feature map and places it in the output matrix [27]. The final pooling layer’s output is flattened into a one-dimensional vector by a flatten layer and delivered to the FC layer as input [28]. It connects the high-level features from the convolutional layers output with the image and, ideally, outputs the accurate classification [27].

## 2. Materials and Methods

### 2.1. Cells and Cell Culture

All experiments were performed using immortalized mouse mesenchymal stem cells (mMSCs) developed by Dr. Tong-Chuan He at the University of Chicago [29]. The cells were cultured in Dulbecco’s modified Eagle’s medium (DMEM) supplemented with 10% fetal bovine serum (FBS) and 1% penicillin/streptomycin at 37 °C in a 5% CO_2_ atmosphere. Exponential cell growth was maintained by passaging the mMSCs every 72 h once they reached ~80% confluence. Cells were counted using an equal volume of single-cell suspension and Trypan blue on the Countess™ II FL (Thermo Fisher Scientific (Waltham, MA, USA)).

### 2.2. mMSC Spheroid Creation and Imaging

**Figure 2:**
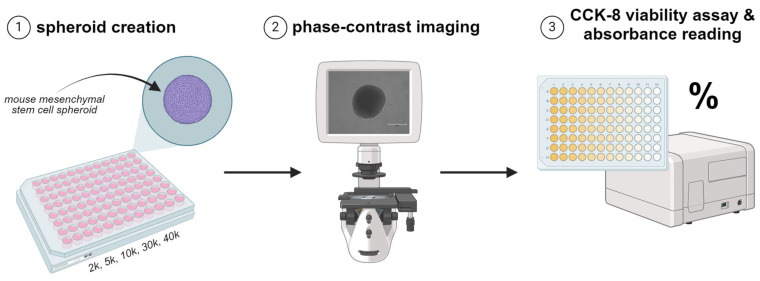
Workflow to establish a comprehensive input dataset: (1) Formation of mMSC spheroids using a 96 WP with varying cell numbers (2 k, 5 k, 10 k, 30 k). (2) Phase-contrast imaging of mMSC spheroids using the EVOS microscope. (3) CCK-8 viability assay using a microplate reader and conversion of the absorbance reading to a viability percentage.

First, the total number of cells needed for a 96-well ULA round-bottom plate (Costar) was calculated by multiplying the desired number of cells per spheroid by 96 wells. The roundness of the well results in cells aggregating at the bottom and forming spherical clusters that become spheroids. It also ensures that a single spheroid is formed per well. To obtain the total volume of cell solution necessary, we divided the total number of cells per plate by the determined cell count after passaging. With a working volume of 200 μL per well, we calculated the total volume of media to add to the cell solution. The calculated volume of media and cell solution was added to a pipetting reservoir, and 200 μL of the solution was manually pipetted into each well of the 96-well plates (96 WPs). The plates were incubated for 72 h at 37 °C in 5% CO_2_. Spheroids made of 2000 (2 k), 5000 (5 k), 10,000 (10 k), 30,000 (30 k), and 40,000 (40 k) cells were created, resulting in diameters ranging from 200 μm to 700 μm (Figure 2). In this paper, spheroid size will refer to the number of cells that make up each spheroid. Spheroid size is measured by the feret diameter, which tends to increase as cell number increases. Typically, 2 k spheroids have a diameter of ~150–200 μm, 10 k spheroids are ~400 μm, and 30 k spheroids are ~500–700 μm. After three days, a phase-contrast image of each spheroid was taken at 20x magnification using the EVOS™ M5000 Cell Imaging System (Thermo Fisher Scientific (Waltham, MA, USA)) (Figure 2).

### 2.3. CCK-8 Viability Assay

One hundred μL were removed from each well of the 96 WP so that only ~100 μL remained in each well. Ten μL (1:10 ratio) of the CCK-8 solution (Sigma-Aldrich (St. Louis, MS, USA)) were added to the medium in each well. The plate was incubated at 37 °C in 5% CO_2_ for four hours. Using the SpectraMax i3x Microplate Reader (Molecular Devices (San Jose, CA, USA)), the plate was shaken for 5 s, and the absorbance of each well was read at 450 nm. A positive control for each spheroid size was made at the time of spheroid creation by seeding 12 wells of a flat-bottom 96 WPs with the appropriate amount of cell solution, medium, and CCK-8 solution, and the absorbance was immediately obtained (Figure 2). A negative control was made by adding 100 μL of medium and 10 μL of reagent and measuring the absorbance. To convert the absorbance value to a viability percentage, Equation (1) was used.

(1) $$percent\;viability=(\frac{sample\;absorbance-negative\;control}{positive\;control-negative\;control})\times 100$$

Equation (1): The equation used to obtain the viability percentage for a spheroid from its absorbance value.

### 2.4. Image Preprocessing and Assembly of the CNN Model

The Python neural networks library Keras from the TensorFlow platform was implemented to build the CNN model. The spheroid images were assigned to either a training (80%), validation (10%), or test (10%) directory, which consisted of 428, 52, and 52 images, respectively. Within each directory, the images were inside folders corresponding to their designated class. The images were rescaled to 1/255 and resized to 244 × 244 pixels. All images were converted from grayscale (one color channel) to RGB (three color channels) using the Python Imaging Library 1.1. 7(PIL). RGB images were necessary to integrate the VGG16 pre-trained model, a CNN model containing weights from a database with 1.4 million images and 1000 classes called ImageNet, into our model. The spheroid images were augmented using the Keras ImageDataGenerator class with the following parameters: rotation_range (20), width_shift_range (0.2), height_shift_range (0.2), horizontal_flip, and vertical_flip. The complete source code is available on GitHub at https://github.com/zyvasheikh/Spheroid-Viability-CNN (7 August 2024).

**Figure 3:**
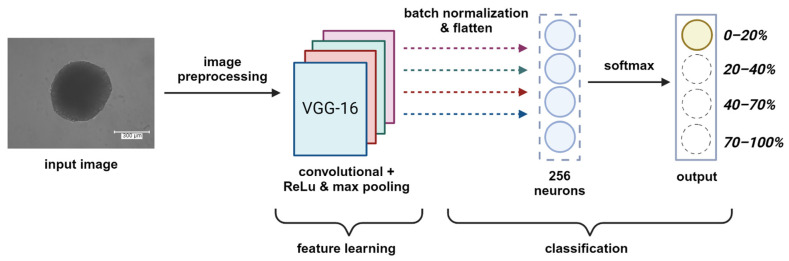
Multiclass classification CNN model architecture. The model receives a preprocessed input image of a spheroid and goes through multiple layers of feature learning, represented by the different colors. It produces an output assigning the image to one of the four possible classes, representing a range of viability percentages for that spheroid.

The model architecture illustrated in Figure 3 begins with leveraging the feature extraction capabilities of the VGG16 base model, which consists of 13 convolutional layers and 5 max pooling layers (Table 1). The 3 FC layers in the network are excluded (include_top = False), allowing customization of the architecture for our specific task. The other layers of the base model were frozen to prevent their weights from being updated during training (layer.trainable = False). In addition to the base model, a batch normalization layer was added to normalize the activations of the previous layer, which helps speed up training and improve convergence. Then, a flatten layer and two FC dense layers were added. The first dense layer consisted of 256 neurons and utilized the ReLU activation function to learn the complex patterns in the data. The second dense layer was the output layer with four neurons, corresponding to the number of classes in the classification task, and a softmax activation function that produces probabilities for each class and allows the model to output a probability distribution over the classes. After constructing the model architecture, it was compiled using the Adam optimizer with a learning rate of 0.0001 and a batch size of 10.

### 2.5. Model Training, Validation, and Test Evaluation

The input for this model was a batch of spheroid images with file names corresponding to their class. The model’s output was one of four classes representing a range of viability percentages: 0–20%, 20–40%, 40–70%, and 70–100%. During training, the model is exposed to the labeled dataset containing input-output pairs and learns to map input data to output predictions by adjusting its parameters, such as weights and biases. Validation assesses the model’s performance on unseen data to ensure it generalizes well and to guide hyperparameter optimization. Categorical cross-entropy was the chosen loss function to evaluate training and validation. It measures the disparity between the predicted class probabilities and the actual class labels for the given input data. The model’s training and validation performance was gauged using accuracy as the metric over 100 epochs with the NVIDIA Tesla K80 graphical processing unit. The training and validation progress was displayed to monitor the model’s performance. The accuracy and loss values were plotted per epoch. The goal was to minimize the loss to ~0.1 and increase the accuracy to ~1 over each epoch. Once the desired performance was achieved on the validation dataset, the model was evaluated on the testing dataset. Testing provides a final, unbiased assessment of the model’s performance on completely unseen data and determines whether the model is suitable for deployment in practical applications. A confusion matrix and a classification report were generated for both validation and testing. The confusion matrix provides a detailed breakdown of the model’s predictions and the actual classes of the data by presenting true positives (TP), true negatives (TN), false positives (FP), and false negatives (FN). The classification report calculates the precision (Equation (2)), recall (Equation (3)), and F1 score (Equation (4)) for each class, as well as the overall accuracy (Equation (5)).

(2) $$precision=\frac{TP}{TP+FP}$$

Equation (2) Precision indicates the proportion of positive predictions made by the model that were correct.

(3) $$recall=\frac{TP}{TP+FN}$$

Equation (3) Recall, or sensitivity, indicates how often the model correctly identifies positive instances from all the true positive samples in the dataset.

(4) $$F1=2\times \frac{precision\times recall\;}{precision+recall}$$

Equation (4) The F1 score provides the harmonic mean of the precision and recall, and it indicates the reliability of the model. An F1 score closer to 1 indicates high precision and recall for a model.

(5) $$accuracy=\frac{TP+TN\;}{TP+TN+FP+FN}$$

Equation (5) The accuracy indicates the proportion of correctly classified instances out of all instances in the dataset.

## 3. Results

### 3.1. Establishment of a Multiclass Classification Model Using Data Cleaning

As we were working with a relatively small dataset, we needed to enhance the model’s capacity to capture important patterns between spheroid size and viability by having the images explicitly depict those features. To do so, we conducted data cleaning, which involved modifying the input dataset by filtering out spheroid images that did not meet the desired size criteria before training the model. As a result, we improved the quality and relevance of the features necessary to emphasize the relationship between spheroid size and viability classes, thus improving the model’s predictive accuracy.

**Figure 4:**
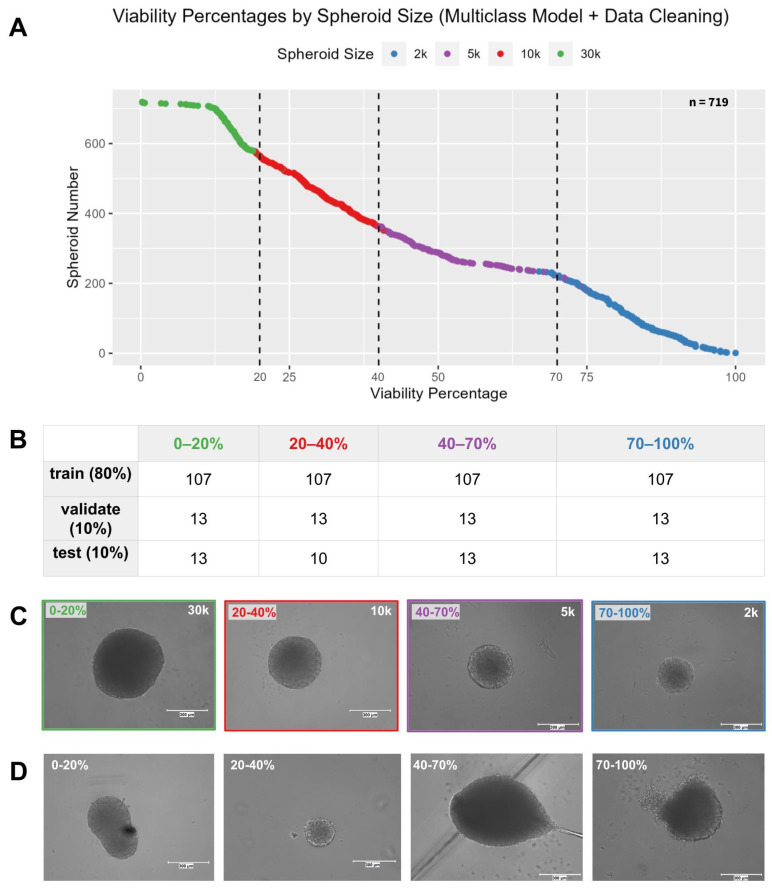
(**A**) Scatter plot of individual spheroids in order of increasing viability percentage after data cleaning, color-coded by their size. Dashed lines dividing the plot at x = 20%, 40%, and 70%. (**B**) The number of spheroid images per class used to train, validate, and test the CNN model. (**C**) Representative spheroid images for each class used to train the CNN model. Scale bar = 300 μm. (**D**) Examples of spheroid images removed from each class due to deformities or incorrect sizing. Scale bar = 300 μm.

To visualize the dataset, we used the ggplot2 package in RStudio to plot the spheroid data points (*n* = 719) in order of increasing viability percentage and color-coded them by spheroid size (Figure 4A). The class boundaries were established approximately where one spheroid size ended and the other began. Therefore, 30 k spheroids were assigned to the 0–20% class, 10 k spheroids to the 20–40% class, 5 k spheroids to the 40–70% class, and 2 k spheroids to the 70–100% class (Figure 4C). The data were cleaned by removing all of the spheroid images that did not fall within the aforementioned sizes corresponding to each class, as well as deformed spheroids (Figure 4D). As a result, each class was composed of images from spheroids of a single size (Figure 4A). All images were augmented. There were 107 images in the training batch, 13 images in the validation batch, and 13 images in the testing batch for each class, resulting in a total of 428 training images, 52 validation images, and 52 test images (Figure 4B). It was compiled using the Adam optimizer with a learning rate of 0.00001. Categorical cross-entropy and accuracy were used to evaluate the model’s training and validation performance over 100 epochs.

### 3.2. Multiclass Classification Model with VGG-16 Architecture Yields High Accuracy, Recall, and Precision

**Figure 5:**
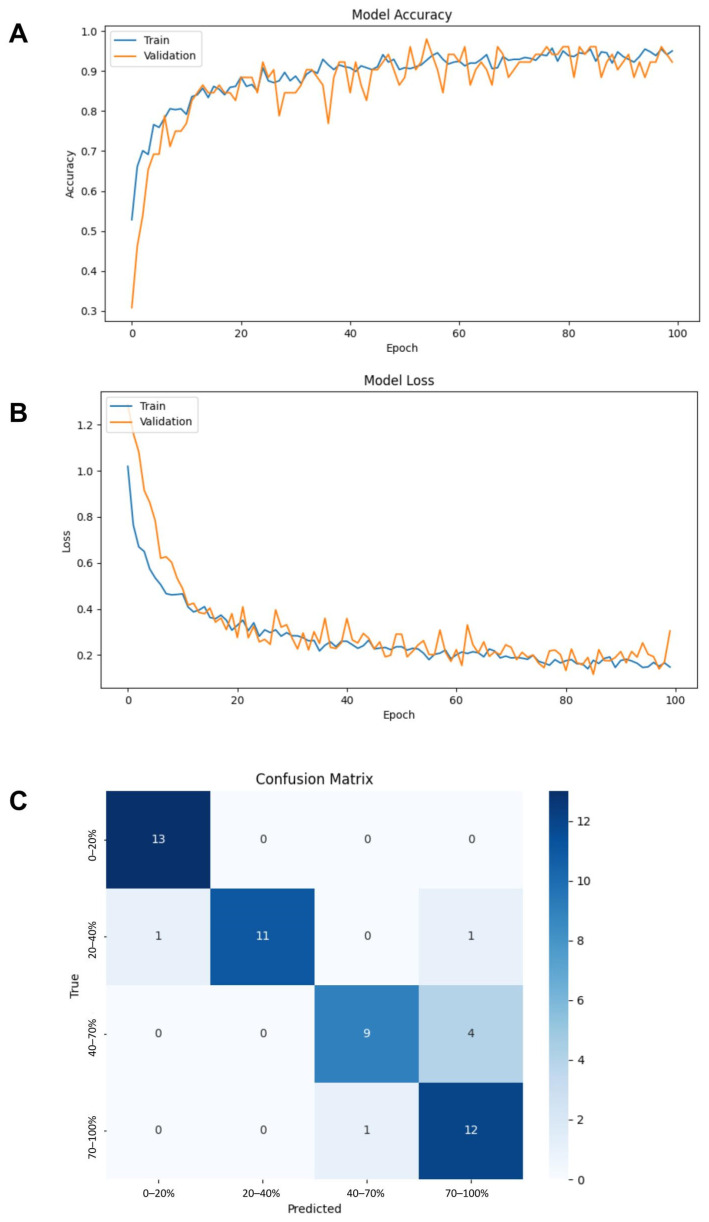
(**A**) Training accuracy and validation accuracy over 100 epochs. (**B**) Training loss and validation loss over 100 epochs. (**C**) Confusion matrix for all classes after training.

Model assembly is as described in Methods. Notably, this model utilizes the VGG-16 pre-trained model architecture and is run using the NVIDIA GPU. After only 12 epochs, the model consistently achieved a training accuracy above 80% (Figure 5A). Although there were slight fluctuations in the validation accuracy, it remained above 75% and reached up to 97% (Figure 5A). Training and validation loss decreased below 0.2 with minimal deviations in the validation loss (Figure 5B). The confusion matrix indicated that the model was predicting all classes, unlike in previous attempts (Figure 5C). It predicted these classes with high precision and recall, demonstrated by the F1 score of 0.96 for the 0–20% class and 0.92 for the 20–40% class (Table 2). The 40–70% class had a precision of 0.90 and a recall of 0.69, so the model correctly predicted positive images 90% of the time but only correctly identified 69% of all actual positive images for this class (Table 2). Inversely, the 70–100% class had a precision of 0.71 and a recall of 0.92, so the model only captured 71% of the positive images for this class, but 92% of the images predicted as positive for this class were correct (Table 2). Additionally, the model had an average accuracy of 87% (Table 2). The model’s high performance on both the training and validation data suggested that overfitting was unlikely. Satisfied with these results, we saved this version of the model to assess its predictions on the test dataset.

**Figure 6:**
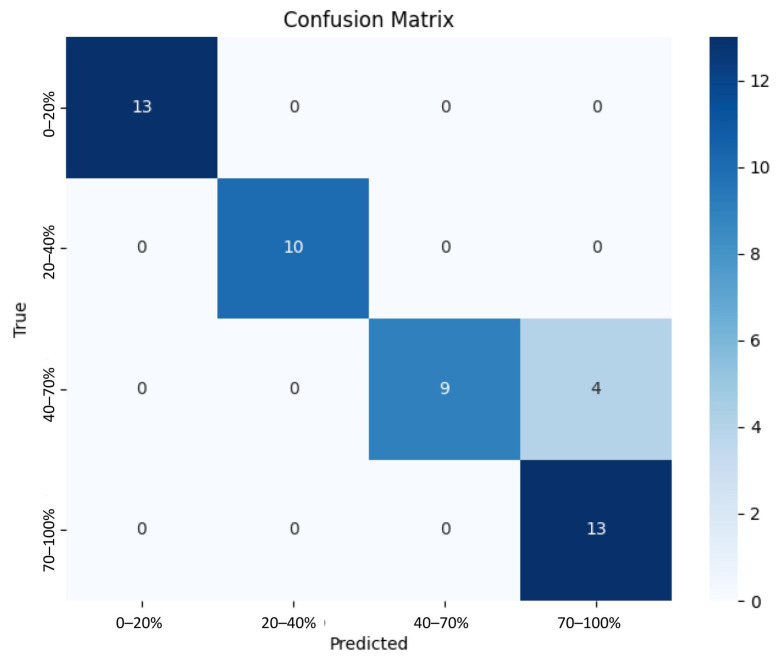
Confusion matrix for all classes after testing.

After testing the model, the confusion matrix again established that the model was successfully predicting all four classes (Figure 6. It was also predicting classes with high precision and recall, with the 1–20% and 20–40% classes having an F1 score of 1.00 and the 40–70% and 70–100% classes having an F1 score of 0.82 and 0.87, respectively (Table 3). For both validation and testing, the 40–70% and 70–100% classes have the lowest F1 scores (Table 3). To note, the test batch for the 20–40% class only had 10 images rather than the assigned 13. The model had an average accuracy of 92% (Table 3).

## 4. Discussion

### 4.1. Small Datasets Are Sufficient to Train CNN Models by Using Transfer Learning and Curriculum Learning

Due to our relatively small dataset size (<1000 images), we prioritized representing a clear relationship between spheroid size and viability percentage to improve the model’s training. We aimed to circumvent the low accuracy associated with using a small dataset by performing feature extraction using the pre-trained VGG-16 model. This process, called transfer learning (TL), involves training and developing a model for a task and then re-using it on a different task to improve performance [30]. TL allows models with smaller datasets to begin with learned features on the significantly larger ImageNet dataset [30]. These features can be adjusted along with the structure of the model to suit the new dataset instead of having to start with random weight initialization [30]. For example, the rarity of abundant and well-labeled data in medical imaging has resulted in a reliance on TL to efficiently train a model on a small dataset [31]. Training a CNN model can be computationally intensive, especially when implementing the VGG-16 model, owing to its deep and complex architecture with many learnable parameters. Conveniently, GPUs can significantly speed up training and predictions by executing operations in parallel and offering significant computational power to run complex models efficiently. This also expedited the process of converting the spheroid images into an RGB format for VGG-16 and allowed the model to run for 100 epochs in only 20 min.

Human-in-the-loop (HITL) machine learning is a type of interaction between humans and ML algorithms in which humans are involved in and have control over the learning process of these models [32]. Curriculum learning (CL) is an approach to HITL training focused on trying to impose some structure on the training set to accelerate and improve learning [32]. Training a model with a curriculum involves starting small, learning easier aspects of the task, and then gradually increasing the difficulty level [32]. There are two motivations for applying CL: to guide and to denoise. CL guides the model by acting as an optimization strategy for CNN models, which first optimizes a smoother and easier version of the problem to reveal the “global picture”, and then gradually considers less smoothing versions [32]. CL denoises the dataset by considering noisy data that are less cognizable as harder examples in the dataset and the cleaner data as the easier ones [32]. This makes training faster, more robust, and more generalizable [32].

The implementation of VGG-16 and CL allowed the model to achieve high accuracy despite the dataset’s limited size. Data cleaning was crucial to implementing CL as it established a dataset that clarified the relationship between spheroid size and viability percentage for training, thereby guiding the model to recognize related features and removing the additional noise that obscured these patterns. The pre-trained model further facilitated effective feature extraction by utilizing weights derived from a more extensive dataset. By combining TL and CL, the CNN model, initially constrained by the dataset’s size, achieved an accuracy exceeding 90%.

### 4.2. High-Performance CNN Model Predicts Spheroid Viability with Limitations

Our multiclass classification model with VGG-16 architecture indicates that DL is a useful tool for optimizing tissue engineering applications. CNNs can be successfully trained to predict a range of viability percentages for a given spheroid image. If this model were to be utilized in a laboratory setting, researchers would only need a phase-contrast image of their spheroid to determine its viability with an accuracy of over 90%. Beyond the model’s high accuracy, it can produce an output within a matter of seconds, starkly contrasting with the hours or days required to run current viability assays. This model also presents a non-invasive method for assessing viability. Instead of subjecting cells to potential damage through confocal imaging, staining, or the disruptive process of dissociating spheroids for FACS analysis, spheroids can remain intact with no additional reagents. This ensures their integrity and allows researchers to confidently utilize them for downstream applications. These results strongly suggest that DL can revolutionize the process of determining spheroid viability and thereby streamline the process of printing cardiac tissue patches. There is minimal research dedicated to leveraging the powerful intersection of artificial intelligence and 3D bioprinting. This paper serves as a vital exploration of these fields and offers an approach that can transform current practices in tissue engineering research. This model could similarly be applied to assessing viability for other purposes, such as following drug exposure.

Nonetheless, the final version of the model only completes the first step of CL by presenting an easier version of the task; this model has yet to be trained on more difficult images. Therefore, the predictive capacity of the current model is not applicable to cases of higher complexity, and the model serves as a proof of concept. Spheroids that do not follow the designated size-viability correlation may be incorrectly classified. Furthermore, there are several restrictions as to what type of input data can be used with this model. The images must be in RGB color format due to the use of VGG-16 in this model. Also, this model is trained solely on images of mMSC spheroids, which can differ from spheroids of other cell types.

Additional metrics can be used to further ensure the model’s high predictive accuracy. The accuracy measure we used does not consider the probability of the prediction [33]. Rather than classification, a ranking using probability estimations could be more suitable [33]. This is possible by finding the area under the curve (AUC) of the Receiver Operating Characteristics (ROC) [33]. The ROC curve compares the classifiers’ performance across the entire range of class distributions and error costs [33]. AUC provides a good “summary” for comparing two ROC curves that do not display a clear dominating relation [33]. It has been found that AUC is statistically consistent and a more discriminating measure than accuracy [33]. Similarly, K-fold cross-validation, in which a dataset is randomly divided into k folds and the model is trained and tested k times on the different folds, is another valuable method to better evaluate the performance of the model. As a result, additional work is required to transform this model from a feasible option to a practical solution.

### 4.3. Limitations in Data Acquisition

Using CCK-8 as the viability assay offered several advantages and drawbacks. CCK-8 allowed us to obtain an individual viability percentage for each spheroid, which initially seemed beneficial as it provided more variability and nuance for the model to train on. It also only took four hours to run, so we could quickly generate hundreds of data points depending on the number of 96 WPs used. Alternatively, FACS analysis requires a minimum of 1 million cells for each test tube, which would require disassociating many spheroids, and necessitates specialized training. However, since the model predicts a range of viability percentages, it would have been satisfactory to obtain a more generalized viability percentage per spheroid size. Additionally, we could not determine whether the CCK-8 reagent could entirely penetrate the core of the spheroid due to the diffusion limit. The cells in the positive control used to calculate the viability percentages were rarely 100% viable, often ranging from 94 to 97%, which further skewed the output. The viability data provided by CCK-8 offered specificity and ease of acquisition but was subject to several inconsistencies. Ideally, an alternative assay that offers high accuracy and efficiency while also providing well-specific data would improve the input data.

### 4.4. Future Directions

To develop a more reliable and scalable model, several changes could be made to the dataset itself and its acquisition. A more accurate assay to obtain viability data, such as FACS, should be implemented, mediating the inconsistencies that stem from CCK-8 while also offering similar efficiency. Significantly more mMSC spheroid data points must be acquired in order to improve the generalization ability of this model to instances of greater variability. Eventually, this model should be further trained to successfully predict viabilities for spheroids of varying cell types, as we do not typically develop our cardiac tissue patches with mMSC spheroids. The feature extraction capabilities of the model can be enhanced by incorporating additional data into the model, such as measurements of mean gray value (indicating cell density), feret diameter, and circularity via ImageJ, to provide more context for predictions. However, it would also be beneficial for the model to not rely entirely on a spheroid’s physical characteristics to predict viability, especially since we are aiming to expand the model beyond the narrow size-viability relationship. For example, fluorescent staining, such as Live/Dead, could be used to visually label the spheroid, providing new features for the model to train on and offering a new way to calculate viability percentages. Another objective would involve increasing the number of classes, thereby refining the predicted range of viabilities and supplying more informative outputs. Overall, this CNN model provides a solid foundation to build an even more comprehensive and nuanced version in the future.

## Figures and Tables

**Table 1:** A model summary outlining each layer of the model and the number of parameters.

Layer	Output Shape	Param. #
vgg16 (Functional)	(None, 7, 7, 512)	14,714,688
batch_normalization	(None, 7, 7, 512)	2048
flatten	(None, 25,088)	0
dense	(None, 256)	6,422,784
dense_1	(None, 4)	1028
Total params:		21,140,548
Trainable params:		6,424,836
Non-trainable params:		14,715,712

**Table 2:** Classification report for all classes after training.

Output Class	Precision	Recall	F1 Score	Support
0–20%	0.93	1.00	0.96	13
20–40%	1.00	0.85	0.92	13
40–70%	0.90	0.69	0.78	13
70–100%	0.71	0.92	0.80	13
accuracy			0.87	52

**Table 3:** Classification report for all classes after testing.

Output Class	Precision	Recall	F1 Score	Support
0–20%	1.00	1.00	1.00	13
20–40%	1.00	1.00	1.00	10
40–70%	1.00	0.69	0.82	13
70–100%	0.76	1.00	0.87	13
accuracy			0.92	49

## Data Availability

The original Python code generated for this study can be found in the Spheroid-Viability-CNN Repository on GitHub at https://github.com/zyvasheikh/Spheroid-Viability-CNN (7 August 2024).
